# Redirection of auxin flow in *Arabidopsis thaliana* roots after infection by root-knot nematodes

**DOI:** 10.1093/jxb/erw230

**Published:** 2016-06-15

**Authors:** Tina Kyndt, Aska Goverse, Annelies Haegeman, Sonja Warmerdam, Cecilia Wanjau, Mona Jahani, Gilbert Engler, Janice de Almeida Engler, Godelieve Gheysen

**Affiliations:** ^1^Department of Molecular Biotechnology, Ghent University (UGent), Coupure links 653, B-9000 Ghent, Belgium; ^2^Department of Plant Sciences, Wageningen University, Droevendaalsesteeg 1, 6708 PB Wageningen, The Netherlands; ^3^INRA, Univ. Nice Sophia Antipolis, CNRS, UMR 1355–7254 Institut Sophia Agrobiotech, 06900 Sophia Antipolis, France

**Keywords:** Arabidopsis, AUX1, auxin, galls, LAX3, PIN, root-knot nematode.

## Abstract

Plant-parasitic root-knot nematodes induce the formation of giant cells within the plant root, and it has been recognized that auxin accumulates in these feeding sites. Here, we studied the role of the auxin transport system governed by AUX1/LAX3 influx proteins and different PIN efflux proteins during feeding site development in *Arabidopsis thaliana* roots. Data generated via promoter–reporter line and protein localization analyses evoke a model in which auxin is being imported at the basipetal side of the feeding site by the concerted action of the influx proteins AUX1 and LAX3, and the efflux protein PIN3. Mutants in auxin influx proteins AUX1 and LAX3 bear significantly fewer and smaller galls, revealing that auxin import into the feeding sites is needed for their development and expansion. The feeding site development in auxin export (PIN) mutants was only slightly hampered. Expression of some *PIN*s appears to be suppressed in galls, probably to prevent auxin drainage. Nevertheless, a functional *PIN4* gene seems to be a prerequisite for proper nematode development and gall expansion, most likely by removing excessive auxin to stabilize the hormone level in the feeding site. Our data also indicate a role of local auxin peaks in nematode attraction towards the root.

## Highlight

Plant auxin efflux and influx proteins redirect the plant hormone auxin towards the feeding site upon root-knot nematode infection in *Arabidopsis thaliana* roots.

## Introduction

Plant roots are constantly challenged by pathogens and parasites present in the rhizosphere. Among them, plant-parasitic nematodes (PPN) inflict considerable damage to a wide range of plant species (Sasser and Freckman, 1986). Because of their economic importance, the best-studied nematodes are the cyst nematodes (CN; *Heterodera* and *Globodera* spp.) and root-knot nematodes (RKN; *Meloidogyne* spp.), which are both biotrophs with sedentary lifestyles.

Second-stage juvenile (J2) nematodes penetrate the plant root at the elongation zone and move towards the root stele, where they manipulate pathways implicated in root development to induce feeding sites called syncytia (for CN) or giant cells (GC; for RKN). GCs induced by RKNs are most commonly derived from parenchymatic cells within the stele that surround the nematode head during parasitism. GC formation starts with the induction of binucleate cells (de Almeida Engler *et al.*, 1999, 2011) followed by repeated rounds of nuclear division, DNA amplification, and cell growth in the absence of cytokinesis. This process, which occurs in five to seven cells around the nematode head, causes them to become multinucleate and hypertrophied, reaching up to 100 times the size of normal root vascular parenchyma cells. Hyperplasia of surrounding cells results in the formation of typical root-knots or galls.

Plant hormones are known to control the regulation of plant growth and development, with transport-dependent auxin gradients triggering the formation of plant organs (Benkova *et al.*, 2003). Auxin plays a major role in plant root development, where it is mainly responsible for cell division, and establishing and maintaining root primordia (De Smet *et al.*, 2010). This hormone is transported from the aerial producing sites towards the root tip through basipetal transport involving influx and efflux transporter proteins. Members of the AUXIN RESISTANT 1 (AUX1) and LIKE AUX1 (LAX) transmembrane protein family govern auxin influx, while the PIN family proteins are responsible for auxin efflux. The spatial and subcellular localization of these proteins drives the auxin flow from source to sink tissues, including plant roots (Wisniewska *et al.*, 2006). Generally, regions with increased auxin levels correlate with the initiation sites of organ primordia in both plant root and shoot tissues (Tanaka *et al.*, 2006).

Interestingly, auxin also plays an important role during the initiation and early development of syncytia induced by CN (Grunewald *et al.*, 2009). An auxin-insensitive tomato (*Solanum lycopersicum*) mutant, diageotropica (*dgt*), was found to be almost completely resistant to these nematodes and the Arabidopsis *axr2/iaa7* mutant, defective in auxin signalling, had a significantly reduced development of CN (Goverse *et al.*, 2000). Chemical inhibition of auxin transport resulted in a reduction of CN development (Goverse *et al.*, 2000) and experiments using the auxin-responsive DR5 reporter revealed that auxin accumulates within young syncytia (Karczmarek *et al.*, 2004). CN manipulate the auxin distribution route (Grunewald *et al.*, 2009), a process which involves enhanced expression of the auxin influx protein AUX1 in the primary syncytial cell (Mazarei *et al.*, 2003) and downregulation of the efflux protein PIN1 (Grunewald *et al.*, 2009). Knowing that nematode infection of *Arabidopsis thaliana pin1* mutants results in a reduced number of cysts and *pin3* and *pin4* mutants support only smaller cysts (Goverse *et al.*, 2000; Grunewald *et al.*, 2009), these auxin export proteins must play important roles in CN parasitism. The Hs19C07 effector of the beet CN *H. schachtii* was shown to interact with the auxin influx protein LAX3 in Arabidopsis roots (Lee *et al.*, 2011). *LAX3* is transcriptionally active within developing syncytia and in cells that are to be incorporated in the syncytium. Although the single *lax3* and *aux1* mutant showed no defects in nematode development, the *aux1 lax3* double mutant and the *aux1 lax1 lax2 lax3* quadruple mutant had significant decreases in female CN numbers at both 14 and 30 days after inoculation (DAI) (Lee *et al.*, 2011).

In contrast to CN, the role of auxin transport on RKN-induced GC formation and development is poorly understood. There is accumulating evidence that auxin also plays a role in the compatible interaction between plants and RKN, but the underlying mechanisms are unknown. For example, application of a synthetic auxin (1-naphthaleneacetic acid) was shown to increase the susceptibility for *M*. *javanica* in otherwise resistant peach plants (*Prunus persica*; Kochba and Samish, 1971). Likewise, application of the natural auxin indole acetic acid (IAA) to tomato roots resulted in a concentration-dependent weight increase of *M. javanica*-induced galls (Glazer *et al.*, 1986). Similar to its resistance to CN, the auxin-insensitive tomato mutant *dgt* does not support RKN development due to an arrest in early feeding site formation (Richardson and Price, 1984). Hutangura *et al.* (1999) observed a strong expression of an auxin-reporter fusion (*GH3pro:GUS*) in *M. javanica*-induced GCs on white clover (*Trifolium repens*) at early (48–72h) time points post inoculation, whereas the signal decreased at later time points (96–120h). Similarly, Karczmarek *et al.* (2004) observed a specific and strong activation of the auxin-responsive *DR5pro:GUS* within the initial feeding cells induced by RKN in Arabidopsis, with the signal most prominent from 18h until 5 DAI, whereas later on the signal decreased. At later time points (7 to 14 DAI), Absmanner *et al.* (2013) reported *DR5pro:GUS* expression in neighbouring cells but not in the GCs in Arabidopsis. Generally, auxin seems to be early and locally accumulating within RKN-induced feeding sites, as in CN-induced syncytia, and thus might also have an important role during gall development. Although strong activity of the *AUX1* promoter within GCs (3 to 14 DAI) has been reported (Mazarei *et al.*, 2003), the role of additional players in auxin transport coordination during gall development is currently unknown.

This gap in current understanding prompted us to study the role of auxin transport governed by AUX1/LAX and PIN proteins during GC development upon RKN infection in Arabidopsis roots. GUS and GFP Arabidopsis reporter lines were used to investigate the redistribution of these proteins during feeding cell development and mutant lines were used to test the importance of the proteins for the establishment of a feeding site. From these data, a model for the redirection of auxin during GC formation is proposed, which was compared to results of former studies regarding the role of polar auxin transport during syncytium development.

## Materials and methods

### Sterilization and sowing of *A. thaliana* seeds for *in vitro* infection experiments

Seeds of *A. thaliana* wild type (ecotype Columbia 0) and En-2 different mutant and transgenic lines were cold-stratified at 4°C for 4 days to synchronize germination. Vapour sterilization of the seeds (50ml H_2_O, 40ml NaOCl, 4.4ml HCl 25%) was performed for 5h followed by further surface sterilization with 70% C_2_H_6_O (ethanol) for 2min and 5% NaOCl for 5min. Seeds were thoroughly rinsed in sterile water.

Approximately 80 seeds were plated for germination on 9cm diameter Petri dishes with Murashige and Skoog medium (MS with vitamins 4.7g/L, 2% sucrose, 0.8% Daichin agar, pH 5.7) and 0.15% plant agar. To allow root development on the surface of the growth medium for ease of transplanting, the Petri dishes were placed vertically in the plant growth chamber at 24°C under a 12h light/12h dark regime. After 5 days, the seedlings were transferred using sterile toothpicks to six-well tissue culture plates (Falcon) containing 4ml of MS medium. Each treatment was replicated 10 times. Growth conditions were maintained at 24°C on a 12h light/12h dark cycle for a period of 7 days to allow sufficient root growth.

### Nematode culture and sterilization

Hatched J2s were collected from tomato roots (pieces of 2–3cm) in a mistifier chamber and subsequently purified with 35% sucrose solution. The juveniles were surface sterilized with HgCl_2_ solution (0.002% Triton X-100 w/v, 0.004% NaN_3_ w/v, 0.004% HgCl_2_ w/v) and rinsed three times in sterile tap water. Prior to inoculation the juveniles were transferred to 0.7% Gelrite solution to allow even distribution of juvenile nematodes and facilitate their movement through the medium.

### Nematode infection assay

Twelve-day-old seedlings of *A. thaliana* were each inoculated with approximately 300 pre-parasitic juveniles (J2) of *M. incognita*. The J2 were equally distributed at the base of the root system using a repetitive pipette (Eppendorf Multipette® Plus). The plants were then transferred to a plant growth chamber operating at 18°C under a 24h dark regime, conditions that favoured nematode infection. We analysed the nematode susceptibility of the plants by counting the number of parasitic J2s, galls, females, and egg masses in roots collected at 3, 7, 35, and 42 DAI. Clean roots were soaked in 10ml of 2.5% NaOCl for 5min to bleach them. To remove residual NaOCl, the roots were rinsed and soaked in tap water. Thereafter, the roots were transferred into acid fuchsin (1:30 acid fuchsin to distilled water) and boiled in a microwave for 30s. After cooling, the excess liquid was drained and the roots were washed with running tap water. Roots were de-stained by boiling in 70% acidified glycerol. Using a binocular microscope, observations and recordings were made of J2s, galls, females, and egg masses. For each line, the number of galls and nematodes in the root system was counted on at least 10 individual plants per experiment. The whole infection experiment was twice independently repeated, giving similar results. In one of these experiments with 10 plants, the developmental stage (in a total of ~400 nematodes per line) and the gall size (in a total of ~250 galls per line) was additionally registered. Data were statistically analysed using SPSS Statistics 20 (IBM^®^), applying a Student’s *t*-test for pairwise comparisons, or ANOVA and Tukey’s test for multiple comparisons of group means.

### Analysis of promoter GUS fusion lines

Different transgenic Arabidopsis plants (Col-0) with the promoters of the auxin efflux genes fused to *GUS* (*PIN1pro:GUS*, *PIN2pro:GUS*, *PIN3pro:GUS*, *PIN4pro:GUS*, *PIN7pro:GUS*) as well as of the auxin influx genes *AUX1pro:GUS* and *LAX3pro:GUS* were grown and after 2 weeks infected with *M. incognita*. GUS staining on non-sectioned galls was done at 3 DAI using the protocol described in Karczmarek *et al.* (2004).

GUS staining on sections of galls formed in promoter fusion lines was done at different time points after nematode inoculation (3, 7, and 14 DAI) as described by de Almeida Engler *et al.* (1999). To avoid diffusion of the GUS precipitate, galls were fixed in 2.0% glutaraldehyde overnight and were then embedded in Technovit 7100, sectioned (3 µm), and microscopically analysed by dark-field optics.

### *In vivo* confocal microscopy

Observation of *PIN1pro:GUS-GFP*, *PIN7pro:GUS-GFP*, *PIN1pro:PIN1-GFP*, *PIN2pro:PIN2-GFP*, *PIN4pro:PIN4-GFP*, and *PIN7pro:PIN7-GFP* expression was performed in nematode-infected Arabidopsis transgenic seedlings. Galls at various time points after infection (7–14 DAI) were dissected from roots and mounted in 5% agar. Thick vibroslices ranging from 50 to 150 μm were made using a Microm HM650V Vibratome (Walldorf). Slices were mounted on microscope slides, a cover slip placed in position, and the slice immediately observed using an inverted confocal microscope (model LSM510 META; Zeiss). Samples were excited with a 488nm argon laser and the GFP-specific fluorescence emission was captured using the lambda spectral mode with a 499–550nm detection bandwidth range. Image analysis and Z-stack projections were done with the LSM 510 software (Zeiss).

## Results

### Spatial distribution and transcriptional regulation of PIN/AUX1/LAX in *M. incognita*-induced feeding sites in Arabidopsis

**Fig. 1.**
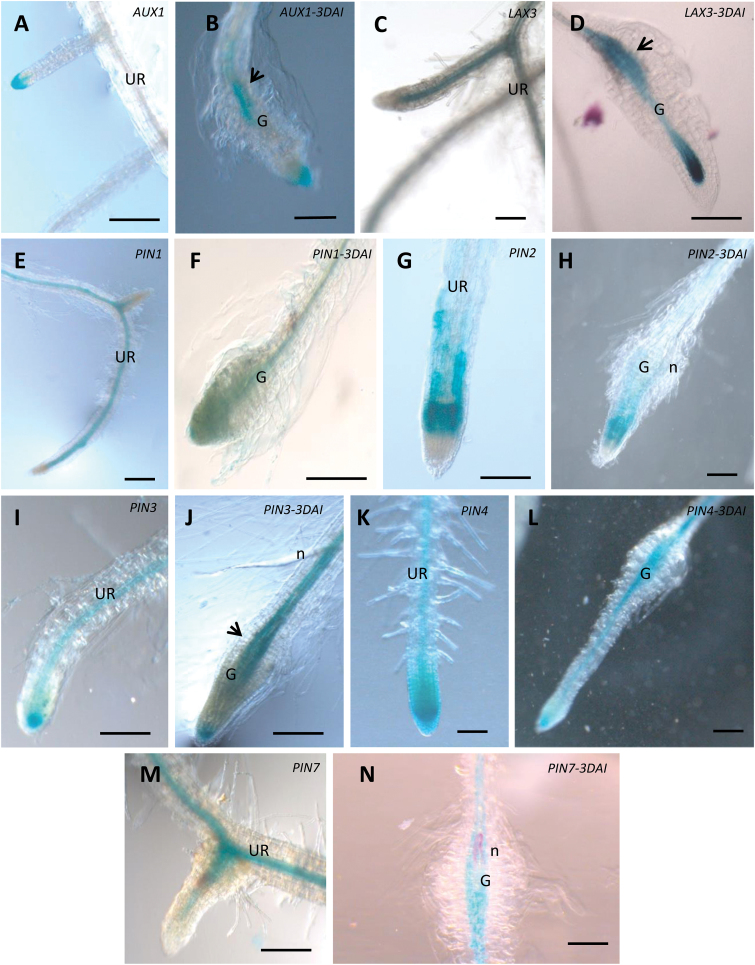
Promoter activity of Arabidopsis auxin transporter genes in uninfected roots (UR) and in young *M. incognita*-induced galls at 3 DAI. (**A, B**) *AUX1pro:GUS* UR and 3 DAI, (**C, D**) *LAX3pro:GUS* UR and 3 DAI, (**E, F**) *PIN1pro:GUS* UR and 3 DAI, (**G, H**) *PIN2pro:GUS* UR and 3 DAI, (**I, J**) *PIN3pro:GUS* UR and 3 DAI, (**K, L**) *PIN4pro:GUS* UR and 3 DAI, (**M, N**) *PIN7pro:GUS* UR and 3 DAI. Arrows point to the basipetal part of the gall where *AUX1*, *LAX3*, and *PIN3* expression is activated. G, gall; n, nematode. Bars = 100 µm.

**Fig. 2.**
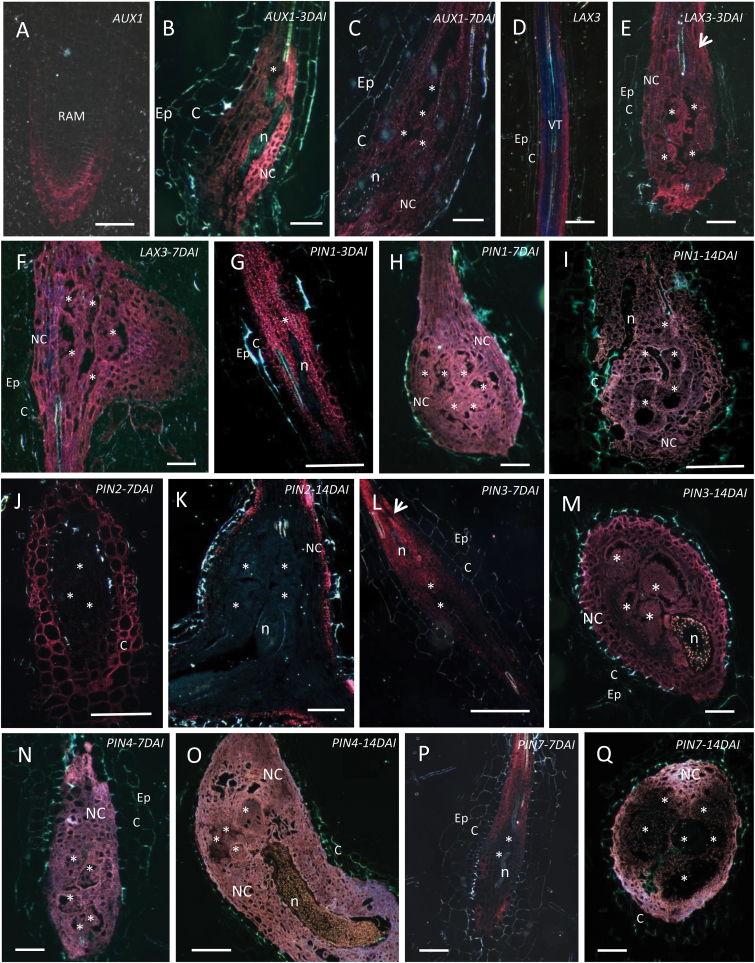
Promoter activity of Arabidopsis auxin transporter genes in uninfected roots (UR) and in *M. incognita*-induced galls at 3–14 DAI. (**A**) *AUX1pro:GUS* in UR and (**B, C**) galls at 3 and 7 DAI, (**D**) *LAX3pro:GUS* in UR and (**E, F**) galls at 3 and 7 DAI, (**G, I**) *PIN1pro:GUS* in galls at 3, 7, and 14 DAI, (**J, K**) *PIN2pro:GUS* at 7 and 14 DAI, (**L, M**) *PIN3pro:GUS* at 7 and 14 DAI, (**N, O**) *PIN4pro:GUS* at 7 and 14 DAI, (**P, Q**) *PIN7 pro:GUS* at 7 and 14 DAI. Arrows point to the basipetal part of the gall where *LAX3* and *PIN3* expression is activated. GUS staining is visualized in red. C, cortex; Ep, epidermis; n, nematode; NC, neighbouring cells; RAM, root apical meristem; asterisk, giant cell. Bars = 50 µm.

It is well known that the activity of different PIN/AUX1/LAX proteins is required for asymmetric auxin distribution in developmental processes, such as leaf, flower, and lateral root initiation (Tanaka *et al.*, 2006). Therefore, *PIN*, *AUX1*, or *LAX* promoter:GUS lines were investigated for their expression during RKN feeding site initiation and development. Previous comparisons with *in situ* hybridization demonstrated that these transgenic lines display similar root expression patterns as the endogenous genes (Friml *et al.*, 2002*a*; Friml *et al.*, 2002*b*; Friml *et al.*, 2003; Abas *et al.*, 2006). *PIN*, *AUX1*, or *LAX3* promoter::GUS-lines were inoculated with the RKN *M. incognita* and promoter activity was investigated at different time points after inoculation. Whole GUS-stained roots and galls are depicted in Fig. 1. For a detailed visualization of tissue and cellular expression, sections are illustrated in Fig. 2. GUS staining of uninfected roots confirmed *AUX1pro:GUS* expression in root tips, mainly at the columella root cap (Figs 1A and 2A). Expression was induced in young galls 3 DAI (Figs 1B and 2B) and 7 DAI (Fig. 2C) and was weak or absent in root tissues surrounding the gall, suggesting an auxin influx during early stages of gall development. In contrast, in uninfected roots *LAX3pro:GUS* expression was absent at the root tip but strong in the root stele (Figs 1C and 2D). During RKN infection, *LAX3* promoter activity was high within young galls (3 DAI and 7 DAI; Figs 1D and 2E, F), suggesting an auxin influx in galls. In addition, both *AUX1pro:GUS* and *LAX3pro:GUS* plants showed slightly stronger staining in cells located at the basipetal side of the developing gall (Fig. 1B, D). The locally induced expression of *AUX1* and *LAX3* reveals that RKN might modulate acropetal auxin transport, most likely to direct enhanced auxin import into the developing feeding site.

*PIN1pro:GUS* was detected in the stele of uninfected roots (Fig. 1E), as well as in young (3 and 7 DAI) and maturing (14 DAI) galls (Figs 1F and 2G, H, I). The expression pattern of the gene encoding the PIN1 auxin efflux carrier, a protein which is responsible for acropetal auxin transport through the root stele towards the root tip (Feraru and Friml, 2008), did not seem to change strongly upon RKN infection in Arabidopsis.

*PIN2pro:GUS*, responsible for basipetal auxin transport (Feraru and Friml, 2008), was expressed in the root cortex and epidermis of uninfected roots, and strongly expressed in the root elongation zone (Fig. 1G). *PIN2pro:GUS* expression was not observed in GCs or neighbouring cells (Fig. 1H), although cortex cells showed GUS staining at different time points after infection (7 and 14 DAI; Fig. 2J, K). This observation could be due to maintenance of the basipetal auxin transport driven by PIN2 in the root cortex, or might indicate that these cortex cells are exporting auxin towards the GCs through the neighbouring cells.

In uninfected roots, *PIN3pro:GUS* showed expression in the root stele and at the root tip (Fig. 1I). At early time points after infection (3 and 7 DAI), high *PIN3* promoter activity was detected in the neighbouring cells at the basipetal side of the gall, but not strongly inside the GCs (Figs 1J and 2L). However, at later time points after infection (14 DAI), *PIN3pro:GUS* expression was clearly observed within GCs and all neighbouring cells (Fig. 2M).

*PIN4pro:GUS* was strongly expressed in the root apical meristem in uninfected roots (Fig. 1K). Whereas *PIN4* promoter activity was weak in the root stele (Fig. 1K), its activity was significantly enhanced within young and mature GCs and neighbouring cells (7 and 14 DAI; Figs 1L and 2N, O).

Similar to *PIN1*, *PIN7pro:GUS* was not expressed in the root tip and GUS staining was observed in the vascular cylinder of uninfected roots (Fig. 1M). *PIN7* expression was suppressed in GCs at all investigated time points (Figs 1N and 2P, Q) but its expression was detected in neighbouring cells (Fig. 2P, Q).

Promoter activity as observed by GUS analyses was confirmed by GFP localization studies performed for one promoter that was found to be active (*PIN1pro:GUS-GFP*) and one that was found to be inactive in GCs (*PIN7pro:GUS-GFP*), and was evaluated in fresh gall slices (Supplementary Fig. S1). Results confirmed the lack of *PIN7* promoter activity at all investigated time points in galls. *PIN1* promoter activity was observed inside GCs until at least 7 DAI, but decreased at later time points. Promoter activity of both *PIN* genes was observed in the root vascular tissue of uninfected roots (Supplementary Fig. S1). Based on these observations as well as Figs 1E, F and 2G–I, we conclude that, for *PIN1*, the basal expression profile is slightly enhanced in young galls (until 7 DAI), whereas *PIN7* expression is specifically suppressed inside the GCs.

### Localization of PIN proteins in *M. incognita*-induced feeding sites in Arabidopsis

**Fig. 3.**
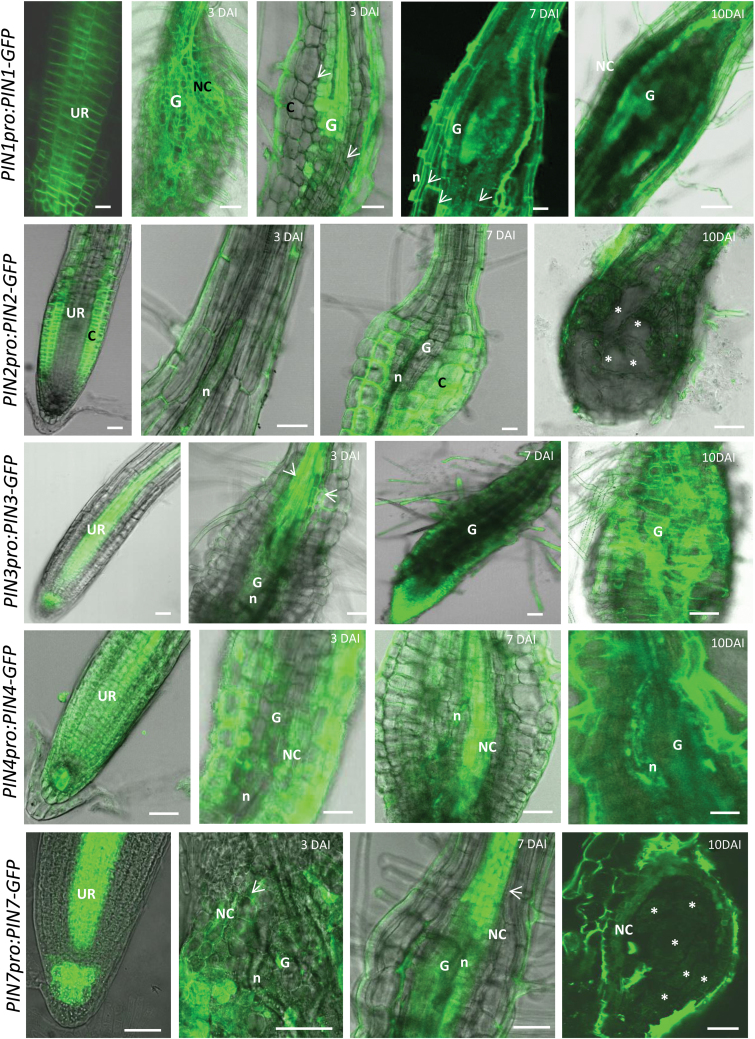
*PIN1pro:PIN1-GFP*, *PIN2pro:PIN2-GFP*, *PIN3pro:PIN3-GFP*, *PIN4pro:PIN4-GFP*, and *PIN7pro:PIN7-GFP* analysis in uninfected roots and in *M. incognita*-induced galls at 3, 7, and 10 DAI in Arabidopsis. Arrows point to the accumulation of PIN1-GFP, PIN3-GFP, and PIN7-GFP at the acropetal side of cells. C, cortex; G, gall; n, nematode; NC, neighbouring cells; UR, uninfected root; asterisk, giant cell. Bars = 25 µm.

Protein localization was analysed using protein–GFP fusion lines of *PIN1pro:PIN1-GFP*, *PIN2pro:PIN2-GFP*, *PIN3pro:PIN3-GFP*, *PIN4pro:PIN4-GFP*, and *PIN7pro:PIN7-GFP* in uninfected roots and in galls at different developmental stages (Fig. 3). PIN1-GFP signal was more intense at the acropetal side of the root cells in uninfected roots and this localization was not changed in cells surrounding the GCs (cortex and epidermal cells). This typical pattern was less clear in GCs due to the high concentration of the PIN1 protein in mainly young GCs (3–7 DAI) (Fig. 3). PIN2 did not accumulate during nematode migration within the root nor at later time points after infection. PIN2 protein was seen in the cortex cells around the GCs, with the same pattern as in uninfected roots (Fig. 3). PIN3 showed localization on the basipetal side of the young gall tissue, as also seen by promoter activity (Figs 1 and 2). Its acropetal localization in cells was not disturbed by the feeding site development. PIN4 was detected around the infecting nematode (3 DAI), with strong expression in GCs and neighbouring cells mainly at 7 DAI, slightly decreasing at 10 DAI. In uninfected roots, PIN7 was typically located in the vascular tissue and in the columella root cap cells (Ferari and Friml, 2008; Fig. 3). In galls, PIN7 was detected in the neighbouring cells, but this protein was absent in GCs (Fig. 3), confirming promoter activity results (Fig. 2 and Supplementary Fig. S1).

### Disruption of auxin transport affects nematode penetration, feeding site initiation, and development

To further investigate the importance of the different auxin influx and efflux proteins for nematode penetration, feeding site initiation, and gall and nematode development, we investigated *pin1*, *pin2*, *pin3*, *pin4*, *pin7, aux1*, and *lax3* mutants and the *aux1 lax3* double mutant. All mutants were infected with RKN *M. incognita*. Preliminary infection experiments on the *pin7* mutant showed no significant differences in gall number (data not shown) and this mutant was therefore not further studied. For all other mutants, the infection success was monitored at different time points after infection: (1) at 3 and 7 DAI to evaluate nematode penetration and feeding site initiation; and (2) at 35 and 42 DAI to monitor gall and nematode development.

### The role of auxin influx/efflux in penetration and feeding site initiation

Acid fuchsin staining of infected plants grown *in vitro* allowed us to monitor nematode penetration within Arabidopsis roots. In wild-type plants at 3 DAI, J2 nematodes had penetrated the plant roots at the elongation zone, and some had already started to initiate a feeding site. By 7 DAI, most nematodes had initiated their feeding sites. In roots of susceptible plants, the number of nematodes was expected to be equal at both time points or slightly higher at 7 DAI (since infection is not synchronized). Because the *pin1*mutant has a different genetic background, it was analysed separately.

**Fig. 4.**
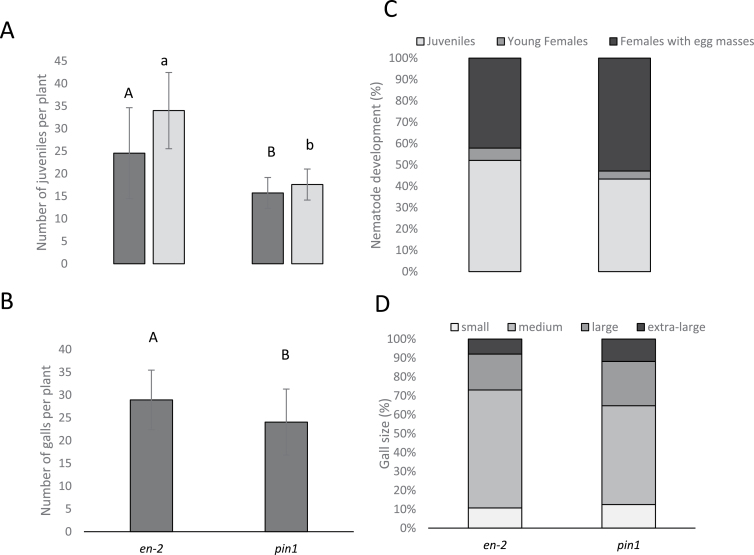
Analyses of *pin1* Arabidopsis mutant and its corresponding wild type En-2, infected by the RKN *M. incognita*. (**A**) Number of J2 per plant, counted at 3 (dark grey) and 7 DAI (light grey). Different letters indicate statistically significant differences based on a Student’s *t*-test (*P* < 0.05), using upper case for 3 DAI and lower case for 7 DAI. (**B**) Number of galls at 35 DAI. Different letters indicate statistically significant differences based on a Student’s *t*-test (*P* < 0.05). (**C**) Developmental stages of the observed nematodes within the galls at 42 DAI, shown as percentages. (**D**) Classification of gall sizes at 35 DAI, shown as percentages. Extra-large (> 2.5mm), large galls (1.5–2.5mm), medium galls (1–1.5mm), and small galls (<1mm). In A and B, bars represent the average ± standard deviation of at least 10 individual plants. The whole infection experiment was twice independently repeated, giving similar results. In C and D, bars represent the percentage of each developmental stage (of ~400 nematodes per line) or gall size (of ~250 galls per line) counted on all 10 individual plants in one infection experiment.

**Fig. 5.**
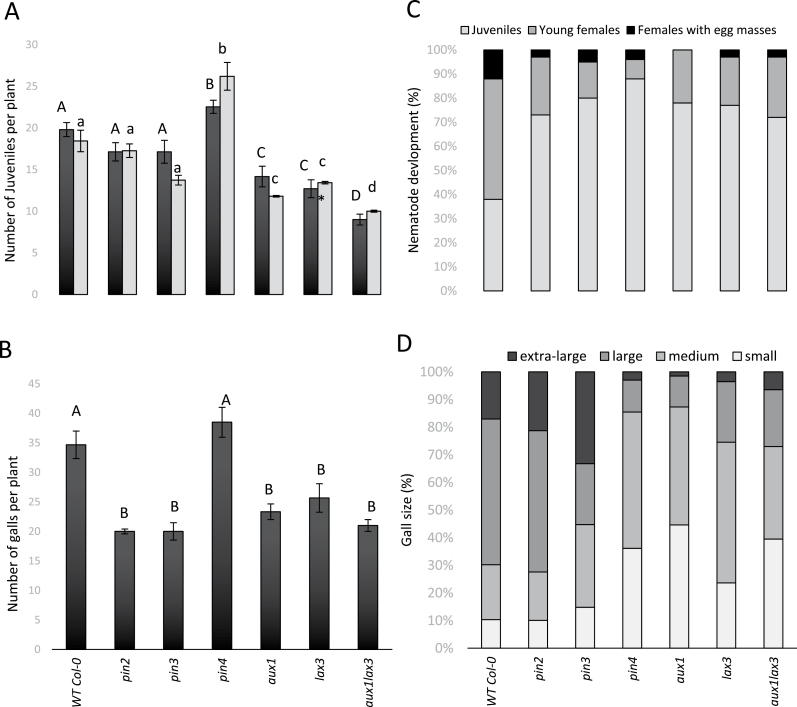
Analyses of *aux1*, *lax3*, *aux1 lax3, pin2*, *pin3*, and *pin4* Arabidopsis mutants infected by the RKN *M. incognita*. (**A**) Number of J2 per plant, counted at 3 and 7 DAI. Different letters indicate statistically significant differences based on ANOVA and Tukey’s test (*P* < 0.05), using upper case for 3 DAI and lower case for 7 DAI. (**B**) Number of galls at 35 DAI. Different letters indicate statistically significant differences (*P* < 0.05). (**C**) Developmental stages of the observed nematodes within the galls 42 DAI, shown as percentages. (**D**) Classification of gall sizes at 35 DAI, shown as percentages. Extra-large (> 2.5mm), large galls (1.5–2.5mm), medium galls (1–1.5mm), and small galls (<1mm). In A and B, bars represent the average ± standard deviation of at least 10 individual plants. The whole infection experiment was twice independently repeated, giving similar results. In C and D, bars represent the percentage of each developmental stage (of ~400 nematodes per line) or gall size (of ~250 galls per line) counted on all 10 individual plants in one infection experiment.

Our infection experiments on the mutant lines (Figs 4 and 5) showed that, compared to the wild-type line En-2, the *pin1* mutant contained significantly fewer nematodes inside the roots at both 3 and 7 DAI (Fig. 4A). The *pin2* and *pin3* mutants (Fig. 5A), by contrast, contained a similar number of nematodes within the roots at 3 and 7 DAI as the wild type. The *aux1*, *lax3* and *aux1lax3* mutants were found to have a 25–50% reduction in juvenile nematodes penetrating the root (analysed at 3 DAI) and during feeding site establishment (analysed at 7 DAI) compared to wild type. For the *pin3* and *aux1* mutant, the number of juveniles was slightly lower at 7 DAI than at 3 DAI, suggesting that feeding site initiation was hampered. Strikingly, the *pin4* mutant showed significantly enhanced nematode penetration and establishment compared to the wild-type plants (Fig. 5A). Taken together, these data suggest a possible increased attractiveness of the *pin4* mutant, whereas the *pin1*, *aux1*, *lax3*, and *aux1 lax3* mutant plants were significantly less penetrated by the nematodes.

### The role of auxin influx/efflux on gall and nematode development

In wild-type plants, gall expansion is typically observed until around 20 DAI (Vieira *et al.*, 2012). After this, nematodes will develop further inside the gall and will lay egg masses on the surface of this root swelling. Gall number and size were evaluated in all mutants (Figs 4B, D and 5B, D), while nematode development within the galls was investigated 42 DAI (Figs 4C and 5C).

Mutants in auxin influx proteins (*aux1*, *lax3*, and *aux1 lax3*) showed low gall number in comparison with wild-type plants (Fig. 5B). This decrease in gall number correlated well with the reduced nematode penetration and feeding site establishment in the roots of these auxin influx mutants (Fig. 5A). Female development within these feeding sites was also hampered (Fig. 5C), and galls in *aux1*, *lax3*, and *aux1 lax3* mutant lines were smaller than in the wild-type Col-0 (Fig. 5D). Remarkably, in the *aux1* mutant line, none of the females produced egg masses at 42 DAI, while the *lax3* mutant and the double mutant allowed egg mass production. Although there was a synergistic effect of *aux1* and *lax3* on nematode penetration (Fig. 5A), *aux1* seems to have the strongest effect on nematode development (Fig. 5C).

Compared to its wild-type en-2, the *pin1* mutant held a significantly lower number of galls (Fig. 4B), which correlated with the reduced number of nematodes inside the *pin1* roots (Fig. 4A). However, nematode development and gall size on the *pin1* mutant line was similar to the wild-type En-2 (Fig. 4C, D).

In the *pin2* and the *pin3* mutants, the number of galls and nematode development were significantly reduced (Fig. 5B, C), although gall sizes were apparently similar to wild-type Col-0 (Fig. 5D). *pin4* mutants held the same number of galls as wild-type Col-0, but we observed a significant inhibition of nematode maturation and gall expansion (Fig. 5B–D). This shows that, although the lack of PIN4 resulted in enhanced nematode penetration and feeding site initiation (Fig. 5A), galls did not expand well (Fig. 5D) and nematode development was strongly hampered by the lack of PIN4 (Fig. 5C).

## Discussion

Herein, we have investigated how influx and efflux proteins are redirecting auxin within the plant root during RKN infection in Arabidopsis. In the data interpretation and discussion, two distinct infection phases were considered: (1) nematode penetration and establishment in the host root (initiation of the feeding site); and (2) feeding site and nematode development. Because our observations strongly suggest that auxin transport plays different roles during those two phases, our results will here be discussed per infection phase.

### The role of auxin during RKN penetration and establishment

The here-reported data on infections of mutant lines validate previous indications that auxin is an important molecule in root attractiveness for nematodes (Curtis *et al.*, 2007). Our results show that *pin4* mutants, reported to accumulate higher auxin levels in the root tip (Friml *et al.*, 2002*a*), are more susceptible to RKN penetration, as seen by the high number of juveniles within the plant host (3–7 DAI). A second argument for the role of auxin in root attractiveness is that the investigated mutants in auxin influx proteins (AUX1, LAX3) and in the polar auxin transporter PIN1 are less susceptible to RKN penetration. These proteins are necessary to direct auxin transport from the source (shoot apical meristem) towards the sink tissues, such as roots where nematodes invade and establish their feeding sites. In *aux1* and *pin1* mutants, the IAA levels within the apical root regions were consistently lower than those found in comparable regions of the wild-type root (Marchant *et al.*, 2002; Zhang *et al.*, 2014). These observations suggest that local auxin maxima at the root tip direct nematode penetration in host roots. It has also been previously suggested that auxin induces changes in the surface cuticle and behaviour of *Meloidogyne* spp., illustrated by the increased stylet thrusting and higher motility (Curtis *et al.*, 2007). High auxin concentrations have also been shown to attract *Aphelenchoides besseyi* nematodes (Feng *et al.*, 2014). An alternative explanation for the observed increased attractiveness of the studied mutant is a potential auxin-induced change in root exudates, which are involved in host location. For example, elevated levels of ethylene were shown to be correlated with decreased host attraction by RKN (Fudali *et al.*, 2013). We cannot, therefore, exclude the possibility that a local increase in auxin upon nematode infection results in disturbances in ethylene production, which ultimately affects host attractiveness. Possible crosstalk between auxin and ethylene in host location is further supported by the Arabidopsis auxin transport mutant *pin2*, which has also been described as the ethylene mutant *eir1* (Luschnig *et al.*, 1998). However, no change in host penetration was observed for the *pin2* mutant in the current study.

### The role of auxin during feeding site and nematode development

**Fig. 6.**
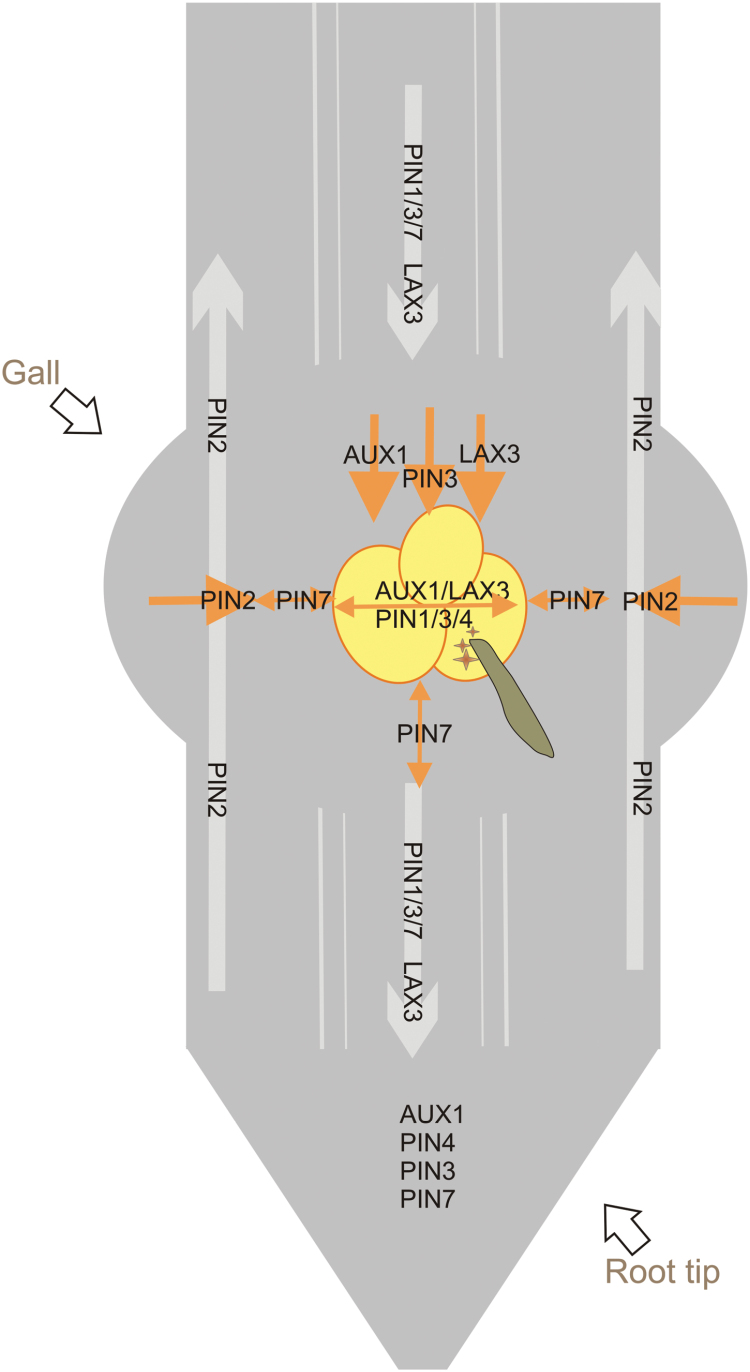
Model of AUX1/LAX3/PIN-mediated auxin transport during nematode feeding site development, based on the 3 and 7 DAI data provided in this manuscript. The giant cells are shown as yellow circles, and the nematode is the green-brown worm. Grey arrows show the direction of the auxin flow in uninfected Arabidopsis roots, and the auxin import/export proteins that are mainly responsible for this flow (based on Feraru and Friml, 2008). Orange arrows show the redirected auxin flow in nematode feeding sites, based on the data provided in this study. n, nematode; brown stars, nematode-secreted auxin. This figure is available in colour at *JXB* online.

Previous research using auxin-responsive promoters showed that auxin accumulates in young GCs, but that this process is transient and the auxin response shifts to neighbouring cells 2–5 DAI (Hutangura *et al.*, 1999; Karczmarek *et al.*, 2004). Auxin inside GCs might be partially derived from *M. incognita* secretions, which have been shown to contain auxin conjugates, albeit in very low quantities (De Meutter *et al.*, 2005). In addition, and probably more important for the reported accumulation of auxin in the GCs, our results provide evidence for a redirected flow of endogenous auxin within the plant during RKN feeding site development. Based on our data, the model depicted in Fig. 6 illustrates this auxin redirection in the RKN-induced feeding sites up to 7 DAI. The normal auxin transport from the shoot apex towards the root tip is shown with grey arrows. The redirected flow during feeding site development is visualized in orange.

GUS analyses showed that at the basipetal region of the gall and in the GCs, *AUX1* and *LAX3* expression were notably induced at 3 and 7 DAI, indicating that AUX1 and LAX3 actively import auxin into the young GCs and their neighbouring cells, which make up the nematode feeding site. *LAX3pro:GUS* expression is also reportedly induced within young syncytia (Lee *et al.*, 2011). For *AUX1*, promoter activity in whole galls and syncytia was comparable to that reported by Mazarei *et al.* (2003), although those authors did not section the feeding sites to visualize expression at the cellular level. Herein, we present the cellular expression pattern of *AUX1* in sectioned galls, revealing GUS expression in GCs as well as in neighbouring cells. Taken together with the observation that the *aux1* and the *lax3* mutants as well as the *aux1 lax3* double mutant contained significantly fewer nematodes, fewer galls, and slower nematode development, we propose that auxin import through AUX1 and LAX3 is required for GC initiation, gall expansion, and, thus, nematode development. In comparison, *LAX3* is transcriptionally active within developing syncytia and in cells to be incorporated in the syncytium. While the single *lax3* and *aux1* mutants showed no defects in CN development, the *aux1 lax3* double mutant and the *aux1 lax1 lax2 lax3* quadruple mutant had significant decreases in female CN numbers (Lee *et al.*, 2011).

The similar expression pattern of *AUX1*, *LAX3*, and *PIN3* strongly suggests that the PIN3 protein facilitates the export of auxin from neighbouring cells at the basipetal side towards the developing GCs. The presence of plasmodesmata between GCs and neighbouring cells (Hofmann *et al.*, 2010) might further facilitate auxin transport. Interestingly, there are some recent insights that show the need for a sequential regulation of LAX3 and PIN3 expression during lateral root emergence. During that process, LAX3-dependent auxin accumulation induces cell wall–modifying enzymes that loosen the cell wall and allow the newly formed lateral root to emerge through the existing root tissues (Swarup *et al.*, 2008; Péret *et al.*, 2013). Mathematical modelling and experimental validation in Arabidopsis roots showed that the interplay between PIN3 and LAX3 can create sharp intercellular gradients in LAX3 expression. The authors suggested that, by expressing *PIN3*, cells can communicate effectively with their neighbours, thereby allowing them to coordinate which of the cells is going to express *LAX3* (Péret *et al.*, 2013). Therefore, considering the similar spatiotemporal expression pattern of *AUX1*, *LAX3*, and *PIN3*, a similar pathway could be expected in galls. During CN infection, *PIN3* gene expression was observed within young syncytia (2 and 5 DAI) (Grunewald *et al.*, 2009). The PIN3-GFP fusion protein revealed a change in the polar localization of PIN3 during syncytium development (Grunewald *et al.*, 2009), whereas at later stages (>5 DAI) PIN3 was more highly expressed in cells neighbouring the developing syncytium. This local expression has been assumed to be important for incorporating neighbouring cells into the growing syncytium (Grunewald *et al.*, 2009). Differently for galls, *PIN3pro:PIN3*-*GFP* analysis showed similar PIN3 localization, at the acropetal side of cells, in both uninfected roots and galls/GCs. This might be explained by the fact that GCs do not fuse with neighbouring cells in galls. Intracellular PIN1 and PIN7 localization was also not changed by gall formation.

During gall initiation and development, GUS analyses and protein localization analyses showed that the activity of *PIN2* and *PIN7* genes as well as protein expression was remarkably absent in the GCs. These genes are also not active in young syncytia (Grunewald *et al.*, 2009). The lack of expression of these two auxin export proteins in GCs probably prevents auxin drainage, hence leading to enhanced auxin levels within GCs. In addition, *PIN2* expression in the cortex and *PIN7* expression in neighbouring cells might be involved in the export of auxin from these cells towards GCs, allowing their proper development and, consequently, nematode maturation. For *pin2*, this hypothesis is supported by functional analysis using the *pin2* mutant lines. Whereas this mutant is equally as susceptible as the wild type to CNs (Grunewald *et al.*, 2009), RKN development was delayed in the *pin2* mutant line, resulting in fewer mature females and egg masses than the wild type. Infection experiments on a *pin7* mutant showed no significant differences in gall number (data not shown) and this mutant was therefore not further studied. *PIN7* expression is clearly suppressed in GCs (Figs 1–4), and this might explain why a complete knock-out does not have an effect on gall formation.

Despite the fact that *PIN1* is expressed in GCs, and a reduced number of nematodes are present inside the mutant roots, only a slight difference in gall number was observed in *pin1* mutant compared to wild-type roots. Gall size and nematode development were not influenced by the *pin1* mutation. This indicates that PIN1 is not needed for gall and nematode development, but seems to be necessary for acropetal auxin transport towards the root tip, where its accumulation could affect nematode attraction. In contrast, *PIN1* expression is downregulated in young syncytia, and *pin1* mutants support significantly fewer and smaller cysts (Grunewald *et al.*, 2009). *PIN1* downregulation most likely is correlated with decreased auxin export from CN-induced syncytia (Goverse *et al.*, 2000; Grunewald *et al.*, 2009). In contrast to syncytia (Grunewald *et al.*, 2009), *PIN1* expression does not seem to be important for GC development.

Interestingly, the *PIN4*-promoter is active in RKN-induced galls, whereas the PIN4 protein is normally mainly expressed at the root quiescent centre, where this protein is known to be regulating auxin homeostasis and patterning through sink-mediated auxin distribution at the root tip (Friml *et al.*, 2002*a*). Although a lack of PIN4 leads to enhanced nematode penetration and feeding site initiation, *PIN4* expression is needed for proper gall expansion and consequently nematode development, as seen by the lower number of mature females (with egg masses) in the *pin4* mutant compared to wild-type galls. Having determined the *PIN4* gene expression and protein levels within GCs and neighbouring cells, this protein might be considered as an important regulator of auxin homeostasis within developing GCs. Similarly, in CN-induced syncytia, the *PIN4*-promoter was shown to be induced at early time points (2 and 5 DAI) during development and cysts were smaller in the *pin4* mutants compared to the wild type (Grunewald *et al.*, 2009).

Even though similarities were observed (e.g. the importance of PIN4 expression in the feeding sites), results obtained in this RKN study show some differences from the data obtained during CN infection (e.g. the importance of PIN1 for syncytia versus PIN2/3 for GCs development) of the same Arabidopsis transgenic/mutant lines (Goverse *et al.*, 2000; Grunewald *et al.*, 2009). Based on the observed differences, we hypothesize that, due to the evolutionary divergence between RKN and CN (Holterman *et al.*, 2009), both sedentary types of nematodes have evolved different strategies to manipulate auxin transport, which also correlates with a different ontogeny, architecture, and cell wall modification pattern in both types of feeding sites. Our data support the idea that a different set of effectors unique for either CN or RKN are most probably involved in the establishment of the nematode feeding site. For example, as far as we know, no ortholog of the *H. schachtii* effector Hs19C07, interacting with LAX3 (Lee *et al.*, 2011), has been found in the RKN secretome. Thus, which mechanisms RKN are using to commandeer auxin distribution in the plant root remain to be investigated. However, as no RKN effector with similar action to Hs19C07 has been found yet, it is equally possible that auxin transport rearrangements arise in response to RKN infection, and are not actively manipulated by the nematode.

Our data conclusively support a model (Fig. 6) in which RKN infection of plant roots affects the auxin distribution patterns during feeding site development. Auxin import at the basipetal side of the gall seems to be induced by the concerted action of AUX1, LAX3, and PIN3. This phenomenon would ultimately lead to auxin accumulation within the GCs induced by *M. incognita.* Local auxin maxima correlate with the initiation sites of organ primordia in both plant roots and shoots (Tanaka *et al.*, 2006) and most likely feeding sites. During plant root development, auxin is responsible for cell division and for the establishment and maintenance of root primordia (De Smet *et al.*, 2010). Moreover, auxin is known to facilitate radial expansion in the root elongation zone (Strader *et al.*, 2010), where RKN infect host roots and initiate their feeding sites, supporting the role for auxin in GC expansion.

## Supplementary data

Supplementary data are available at *JXB* online.

Figure S1. Promoter activity of Arabidopsis *PIN1pro:GUS-GFP* and *PIN7pro:GUS-GFP* in uninfected roots and in *Meloidogyne incognita*-induced galls at 7–14 DAI.

Supplementary Data
