# Supplementary material for: Redirection of auxin flow in Arabidopsis thaliana roots after infection by root-knot nematodes
Source: J Exp Bot. 2016 Jun 15;67(15):4559–70. doi: 10.1093/jxb/erw230 (PMC4973730; doi:10.1093/jxb/erw230)
Supplement: Supplementary Data [file supp_67_15_4559__index.html]

Redirection of auxin flow in Arabidopsis thaliana roots after infection by root-knot nematodes — Redirection of auxin flow in Arabidopsis thaliana roots after infection by root-knot nematodes — Supplementary Data 

# Redirection of auxin flow in *Arabidopsis thaliana* roots after infection by root-knot nematodes

## Supplementary Data

Data files

- supplementary\_figure\_S1.pdf - Supplementary Data
