# Supplementary material for: Redirection of auxin flow in Arabidopsis thaliana roots after infection by root-knot nematodes
Source: J Exp Bot. 2016 Jun 15;67(15):4559–70. doi: 10.1093/jxb/erw230 (PMC4973730; doi:10.1093/jxb/erw230)
Supplement: Supplementary Data [file supp_erw230_supplementary_figure_S1.pdf]

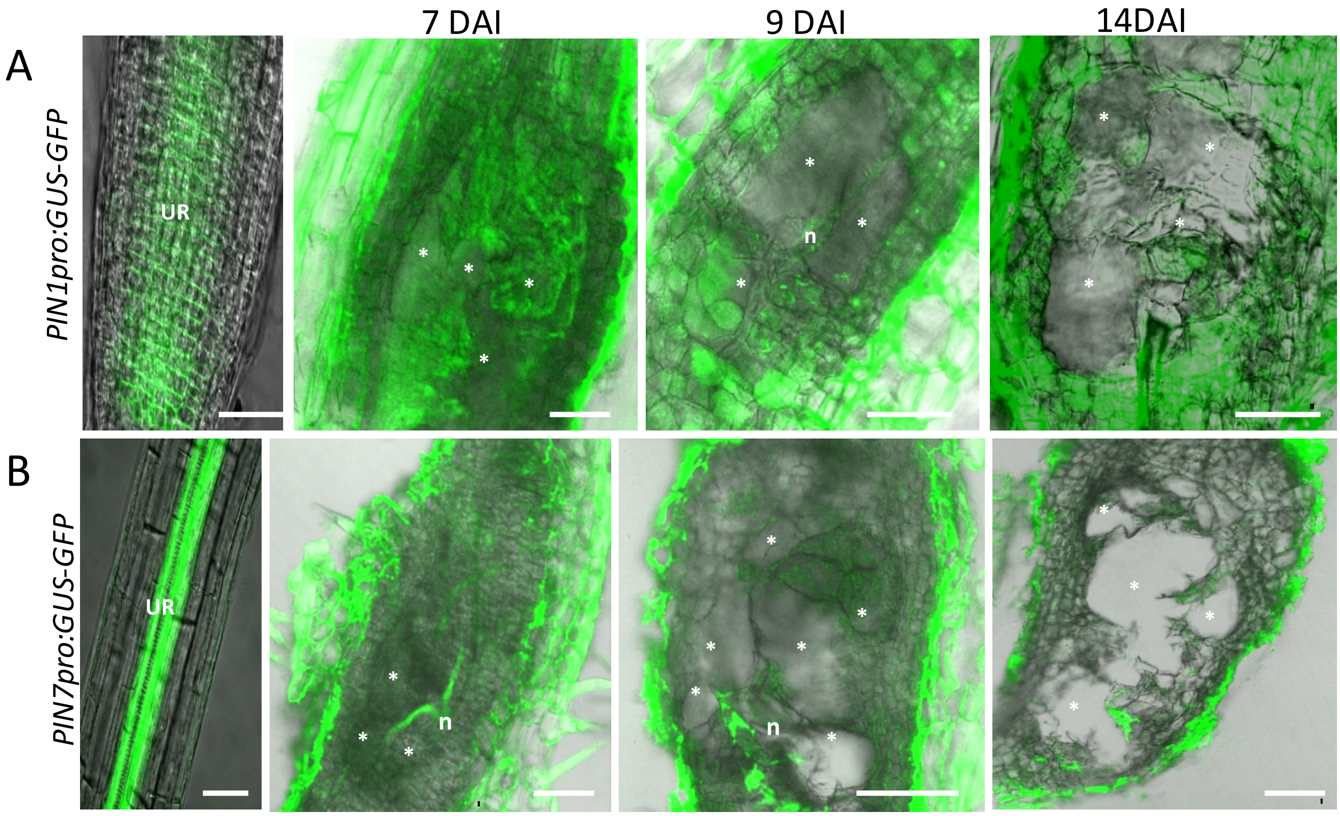

Supplemental Figure 1: Promoter activity of Arabidopsis *PIN1pro:GUS-GFP* and *PIN7pro:GUS-GFP* in uninfected roots and in *Meloidogyne incognita*-induced galls at 7 to 14 days after infection (DAI). (A) *PIN1pro:GUS-GFP* in an uninfected root, and in galls at 7, 9 and 14 DAI. (B) *PIN7pro:GUS-GFP* in an uninfected root, and in galls at 7, 9 and 14 DAI. UR, uninfected root; G, gall, n, nematode. Bars = 25µm.
